# Supplementary material for: Time‑resolved multi-omic analysis of paclitaxel exposure in human iPSC‑derived sensory neurons unveils mechanisms of chemotherapy‑induced peripheral neuropathy
Source: Cell Death Dis. 2026 Feb 10;17(1):211. doi: 10.1038/s41419-026-08445-2 (PMC12921266; doi:10.1038/s41419-026-08445-2)
Supplement: Supplementary file 15 — Dataset 1. [file 41419_2026_8445_MOESM15_ESM.zip › CDD_Open_Data/04_RNA_Seq/Timeline_panel_plots_etc_2023-01-23_1609.zip!/Panel_Plots_etc/Evidence_Plots/post5d/evidence_plot_standard contrasts_msigdb_reactome_5d_PTX_vs_DMSO_ID6_M7223_REACTOME_TRAFFICKING_OF_AMPA_RECEPTORS.pdf]

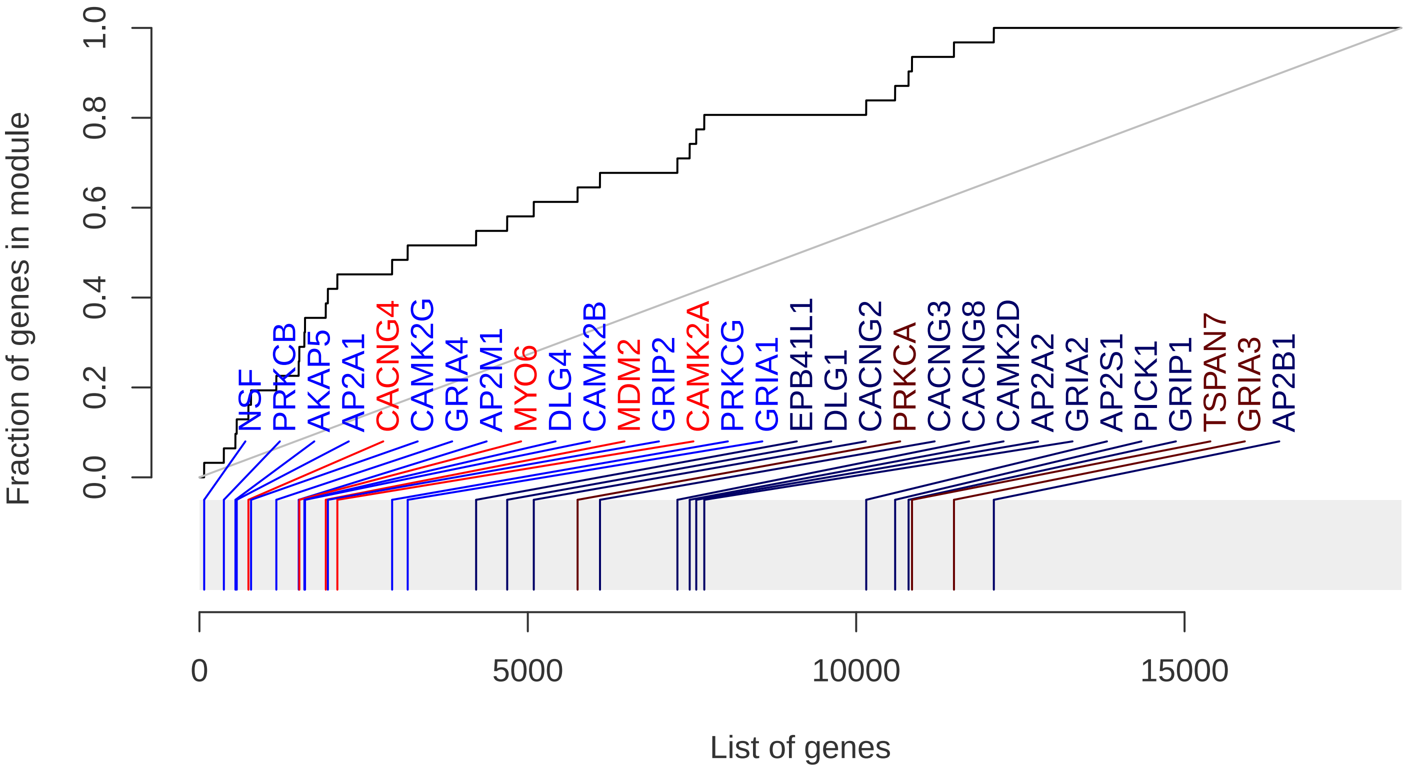

Fraction of genes in module
0.0
0.2
0.4
0.6
0.8
1.0
NSF
PRKCB
AKAP5
AP2A1
CACNG4
CAMK2G
GRIA4
AP2M1
MYO6
DLG4
CAMK2B
MDM2
GRIP2
CAMK2A
PRKCG
GRIA1
EPB41L1
DLG1
CACNG2
PRKCA
CACNG3
CACNG8
CAMK2D
AP2A2
GRIA2
AP2S1
PICK1
GRIP1
TSPAN7
GRIA3
AP2B1
0
5000
10000
15000
List of genes
